# Identifying factors contributing to kinesiophobia in kidney transplant patients based on the fear-avoidance model: a qualitative study

**DOI:** 10.3389/fpsyg.2026.1844608

**Published:** 2026-05-29

**Authors:** Delong Jiang, Tiantian Chang, Na Hu

**Affiliations:** 1 Department of Vascular and Thyroid Surgery, The First Affiliated Hospital of China Medical University, Shenyang, China; 2 Department of Cardiovascular Surgery, China-Japan Friendship Hospital, Beijing, China; 3 Transplantation and Hepatobiliary Department, The First Affiliated Hospital of China Medical University, Shenyang, China

**Keywords:** fear-avoidance model, influencing factors, kidney transplantation, kinesiophobia, qualitative research

## Abstract

**Background:**

An appropriate and scientific exercise plan for kidney transplant patients is of vital importance for the recovery of the transplanted kidney. The main objective of this study is to explore the factors related to kinesiophobia in kidney transplant patients.

**Methods:**

This study adopted the descriptive phenomenological qualitative research method and selected 13 patients who underwent kidney transplantation (Tampa scale for kinesiophobia, TSK-11>17) in a tertiary hospital in Northeast China. The data collection was conducted using the semi-structured one-on-one interview method, and the text data were analyzed using the Colaizzi seven-step analysis method.

**Results:**

This study identified a total of 4 themes and 11 sub-themes, namely: physiological factors (decreased body function, increased sensitivity to post-operative discomfort); psychological factors (decreased exercise self-efficacy, excessive vigilance towards the transplanted kidney, low self-esteem and feelings of stigma); support system factors (insufficient guidance from medical staff, lack of companionship from family or friends, scarcity of exercise rehabilitation resources); and environmental factors (adverse weather conditions).

**Conclusion:**

The kinesiophobia of kidney transplant patients can affect the recovery of the transplanted kidney. Medical researchers should identify the influencing factors of kinesiophobia in kidney transplant patients as early as possible and take targeted intervention measures to enhance the participation of patients in exercise rehabilitation.

## Introduction

Chronic kidney disease (CKD) is a significant public health issue worldwide. Studies have shown that the number of new cases of chronic kidney disease worldwide has increased from 7.8 million in 1990 to 18.99 million in 2019 (Ying et al., 2024). Among Asian countries, China has the largest number of people suffering from chronic kidney diseases, approximately 159.8 million (Liyanage et al., 2022). According to the sixth round of the China Chronic Disease and Related Risk Factor Surveillance conducted in 2018 ~ 2019, the proportion of Chinese adults with CKD at stage 3 and stages 4 ~ 5 was 25 and 1.8%, respectively (Wang et al., 2023). As chronic kidney disease progresses gradually, it will reach the stage of end-stage kidney disease (ESKD). Currently, kidney transplantation surgery is one of the more effective treatment methods for ESKD besides dialysis. However, patients after kidney transplantation still face many difficulties and challenges (GBD 2023 Kidney Failure with Replacement Therapy Collaborators, 2025).

Studies have shown that after kidney transplantation, patients may experience various negative events, such as low muscle mass, physical fatigue, severe financial burden, and cardiovascular events (Zelle et al., 2016). Therefore, patients may believe that exercise might pull on the transplanted kidney and increase the pressure on the new kidney, they choose to reduce physical activity or avoid exercising altogether and this condition is known as kinesiophobia (Liu et al., 2021). Some studies have shown that patients who have undergone kidney transplants in the early stage of recovery are afraid to exercise due to concerns that their wounds have not fully healed properly (Billany et al., 2022). Chinese scholars have found that the kinesiophobia rate among kidney transplant patients is 62.6%, and it is influenced by factors such as age, educational level, post-operative time, fatigue and depression, and self-efficacy in managing chronic diseases (Tong et al., 2025).

Some researchers have found that 69% of kidney transplant patients are lacking in physical activity, and this is influenced by factors such as family income, educational level, smoking, obesity, and the length of post-operative hospital stay (Sertorio et al., 2024). The 2021 Clinical Practice Guidelines for Chronic Kidney Disease of the British Kidney Society recommend that patients without contraindications for kidney transplantation should be encouraged to engage in at least 150 min of moderate-intensity exercise or 75 min of exercise per week, which can help reduce all-cause mortality, lower obesity, and decrease the occurrence of osteoporosis and cardiovascular diseases (Rovin et al., 2021). A 1-year longitudinal follow-up study found that only 25% of kidney transplant patients met the recommended activity levels as per the guidelines, and their physical activity levels were significantly lower than those of patients with other chronic diseases (Dontje et al., 2014). Some studies have suggested that regular physical activity on a weekly basis for kidney transplant patients may reduce the risk of cardiovascular diseases and prevent the deterioration of the transplanted organ function (Totti et al., 2020). Some scholars have pointed out that the incidence of cardiovascular diseases in kidney transplant patients after surgery is 35% ~ 40%, which is closely related to insufficient physical activity and is also one of the causes of their postoperative deaths (Lethem et al., 1983). If the transplanted kidney loses its function, the patient will once again return to the dialysis stage, which will impose a heavy burden on public health, organ transplantation allocation and supply–demand, as well as family finances. Therefore, it is of great significance to explore the factors related to kinesiophobia in kidney transplant patients. A safe and progressive exercise plan will have a positive impact on the long-term quality of life of kidney transplant patients.

At present, the research on kinesiophobia among Chinese kidney transplant patients mainly focuses on quantitative studies. However, qualitative studies from the perspective of patients in this cultural context regarding kinesiophobia are still lacking. This will limit researchers’ ability to fully understand the fundamental causes of patients’ kinesiophobia. Therefore, in this study, the “fear-avoidance model” was adopted as the framework for designing the interview outline and theoretical basis of the qualitative research (Lethem et al., 1983). The fear-avoidance model was initially proposed by Lethem in 1983 to explain the avoidance of physical activity and reduction of activity behaviors triggered by painful stimuli, which is influenced by psychological, external environment, painful experiences, and susceptibility factors (Lethem et al., 1983). As this theoretical model has gradually developed, it has currently been applied to patients with cardiovascular diseases and fractures, and it has pointed out the viewpoint that kinesiophobia can affect the postoperative recovery of patients and increase the risk of complications (Xu et al., 2025; Chen et al., 2025; Ma et al., 2025). In China, the period of 6 months after kidney transplantation is the peak period for infections. Doctors advise patients not to return to work or go to crowded places (Liu et al., 2021). Therefore, this study focuses on kidney transplant patients within 6 months after the operation as the research subjects. However, no studies have been conducted on kinesiophobia among kidney transplant patients in China. Therefore, this study conducted qualitative research on the influencing factors of kinesiophobia in Chinese kidney transplant patients based on the fear-avoidance theory, aiming to provide a reference for clinical practice in formulating intervention strategies.

## Methods

### Design

This study adopted a descriptive phenomenological qualitative research method to describe the influencing factors of kinesiophobia in kidney transplant patients after surgery. The investigation report of this study complies with the Standards for Reporting Qualitative Research (SRQR) requirements (O’Brien et al., 2014).

## Materials and methods

From June 2025 to September 2025, patients who met the inclusion criteria for kidney transplantation were recruited at the outpatient department of the organ transplantation section of a tertiary grade A hospital in Northeast China. The maximum variance sampling method in the purpose sampling approach (based on factors such as the age, gender, marital status, and educational level of kidney transplant patients) was used to select the respondents to ensure information diversity. The sample size was determined based on the principles of adequate data and saturation. When no new themes emerged during the data analysis, one more respondent was added until no new themes appeared.

Inclusion criteria: (i) Age ≥ 18 years old; (ii) First kidney transplantation surgery, with the time post-operation ranging from 1 month to 6 months; (iii) A score of more than 17 points on the Kinesiophobia Shortened Tampa Scale (TSK-11) can be diagnosed as kinesiophobia (Woby et al., 2005). It consists of 3 dimensions and 11 items, including activity behavior, activity cognition, and activity attitude. It uses the Likert 4-point rating scale, with a score range of 11 to 44 points. The higher the score, the greater the patient’s degree of Kinesiophobia. In this study, the Cronbach’s *α* coefficient of the scale was 0.856. It has been culturally adapted and used in Chinese kidney transplant patients and has good reliability and validity. (iv) Clear consciousness, with the ability to read and communicate, and willing to participate in the research to describe the relevant experiences.

Exclusion criteria: (i) Patients who have undergone two or more combined organ transplants simultaneously; (ii) those with severe postoperative complications (such as severe recurrence of renal failure, critical condition of severe heart failure, history of myocardial infarction, severe chronic obstructive pulmonary disease, severe rejection reaction, malignant tumor, severe physical and mental disorders, musculoskeletal or neurological disorder that may increase the level of kinesiophobia in the subject); (iii) patients with limb deficiencies, lower limb fractures, unable to participate in exercise training and are difficult to cooperate. A total of 13 participants were involved in the relevant interviews of this study. Their average age was 38.54 (20 to 58). Among them, 5 were female kidney transplant patients. The time since transplantation ranged from 1 to 6 months. Seven of the participants were in hemodialysis status before the surgery, and only 2 had returned to their work positions (Table 1).

**Table 1:** Characteristics of participants (*n* = 13).

No.	Age	Gender	Marital status	Education	Religion	Return to work/Study	Pre-operative dialysis type	Post-transplantation time
P1	26	Women	Unmarried	Undergraduate	No	No	Hemodialysis	1 months
P2	25	Man	Unmarried	Junior college	No	Unemployed	Hemodialysis	3 months
P3	48	Man	Married	undergraduate	Buddhist	No	Undialyzed	1.5 months
P4	43	Women	Married	High school	No	No	Peritoneal dialysis	5 months
P5	52	Man	Divorced	Technical secondary school	Christianity	No	Hemodialysis	4.5 months
P6	32	Man	Divorced	Undergraduate	No	Yes	Hemodialysis	6 months
P7	20	Man	Unmarried	Undergraduate	No	No	Hemodialysis	3.5 months
P8	45	Women	Married	High school	No	Unemployed	Peritoneal dialysis	2 months
P9	40	Women	Married	Junior college	Buddhist	No	Undialyzed	1.5 months
P10	50	Man	Married	Technical secondary school	No	No	Peritoneal dialysis	2.5 months
P11	58	Man	Spouse deceased	Junior college	Buddhist	Retirement	Hemodialysis	4 months
P12	34	Man	Married	Undergraduate	No	Yes	Peritoneal dialysis	5.5 months
P13	28	Women	Unmarried	Postgraduate	No	No	Hemodialysis	6 months

### Ethical considerations

This study was conducted following the guiding principle of the Declaration of Helsinki, and it was approved by the First Affiliated Hospital of China Medical University (Approval number: No.2025.294). All organ donation procedures are carried out under the supervision of the Red Cross Society and strictly in accordance with the Helsinki Declaration. The organ donation behavior in this study was conducted in accordance with the Declaration of Istanbul. Before the study started, the researcher himself introduced the purpose and method of the study to the respondents, explained the possible risks and benefits during the research process, informed them that the interview process would be recorded, and obtained their voluntary signed informed consent form. The names and relevant personal information of all respondents were reported in alphabetical order to fully protect the privacy of the respondents.

### Rigor and research characteristics

There are four key aspects that ensure the quality control and rigor of qualitative research: credibility, transferability, reliability, and confirmability. To ensure the consistency of the research results, all interviews were conducted by the same researcher, who holds a doctorate in nursing and has 6 years of clinical nursing experience. Firstly, to ensure the credibility of the research results, this study adopted a half-structured interview method, which fully ensures that the respondents can fully describe their experiences of kinesiophobia. The purpose of this study is to determine the transferability of the research results to similar phenomena, so a comprehensive description of the participants’ experiences was provided. Additionally, to ensure the reliability and confirmability of the research, the researcher used a unified interview outline during each interview, and a reflection memo was written promptly on the day of the interview to reduce the influence of preconceived notions on the research results and to avoid getting overly involved in the research due to relevant work experience. All researchers conducted self-reflection and promptly provided feedback on the research results to the participants.

### Data collection

The main research tool was the researcher. Other tools included computers, voice recorders and diaries. A semi-structured interview method was adopted. Before the interview, the researcher initially drafted the interview outline based on the fear-avoidance model and consulted relevant literature. Then, they consulted experts in the field of kidney transplantation to ensure the rigor of the questions. Finally, they discussed with the members of the group to form the initial version of the interview outline. Subsequently, one kidney transplant patient who met the inclusion criteria was selected for a pre-interview. Based on the feedback, the content of the interview outline was revised, and finally the final version of the interview outline was formed (Table 2). During the formal interview, only the interviewee and the researcher were present at the research site. The interview location was chosen in a quiet and clean room, with masks and disinfection supplies prepared. Air disinfection using a disinfection machine was carried out before and after each interview to prevent infection.

**Table 2:** Interview outline of this study.

No.	Interview content
1	How are you feeling after the kidney transplant surgery?
2	What are your thoughts on the exercise rehabilitation after kidney transplantation?
3	Have you ever tried to do any activities or exercises after the kidney transplant surgery?
4	Have you experienced any fear or avoidance of physical activity after your kidney transplant surgery? Why?
5	What factors do you think affected your daily exercise after the kidney transplant surgery?
6	Do you think exercise is beneficial to you? Why do you have this feeling?
7	Have any medical staff explained to you the relevant knowledge about post-operative exercise?
8	Do you have any additional information to add?

Each interviewee was interviewed by the same researcher to ensure the consistency of the research results. Before each formal interview, the researcher asked about the health status after kidney transplantation and showed full concern for the interviewee, establishing trust with them. Throughout the interview, the sequence of interview questions would be appropriately adjusted. In addition to recording the text materials with a recording device, during the interview, a notebook would be used to describe the interviewee’s body language and facial expressions. After the interview, the researcher would reflect on it themselves to supplement the non-verbal data. The interview lasted approximately 39–48 min.

### Data analysis

Within 24 h after each interview, the interview recordings will be transcribed into written materials. All the text materials will be password-protected and backed up. The NVivo 12.0 software will be used to establish a database. The Colaizzi seven-step analysis method will be adopted to analyze the text materials on the topic of sports fear. The text materials will be independently analyzed by two researchers. The analysis steps include: Firstly, reading the text information multiple times to fully understand the meaning of the text materials. Secondly, identifying the important information and statements in the text data. Thirdly, forming meaningful concept expressions and coding. Fourthly, classifying the concepts, including themes and sub-themes; fifthly, fully integrating and detailing the research results; sixthly, providing a comprehensive description of the structure of this phenomenon. Finally, the obtained research results will be shared with the interviewees. The research results will be fully verified through the feedback from the interviewees. If there is a dispute between the two researchers, it will be discussed and determined with another researcher in the research group.

## Results

### Main them and sub-themes

This study identified a total of 4 main themes and 9 sub-themes, namely: physiological factors (decreased body function, increased sensitivity to postoperative discomfort), psychological factors (decreased exercise self-efficacy, excessive vigilance towards the transplanted kidney, low self-esteem and feelings of stigma), support system factors (insufficient guidance from medical staff, lack of companionship from family or friends, scarcity of exercise rehabilitation resources), environmental factors (unfavorable climatic conditions) (Table 3).

**Table 3:** Themes and sub-themes.

Themes	Sub-themes
Physiological factors	Reduced body function
	Increased sensitivity to postoperative discomfort
Psychological factors	Excessive vigilance towards transplanted kidneys Decrease in exercise self-efficacy
	Self-esteem and the sense of social stigma
Supporting system factors	Insufficient guidance from medical staff
	Lack of company from family or friends
	Insufficient resources for sports rehabilitation
Environmental factors	Unfavorable weather conditions

### Physiological factors

#### Reduced body function

Patients undergoing kidney transplantation typically experience a protracted medical journey. Their treatment process encompasses the arduous phase of pre-operative dialysis, the stage of waiting for and preparing for kidney transplantation, and the post-transplantation rehabilitation phase. The physical functions of patients undergoing kidney transplantation usually remain lower than those of healthy individuals after the surgery. The kidney transplant patients interviewed in this study experienced symptoms such as fatigue, weakness and lack of physical strength in the early postoperative period. These discomforts also led to the development of kinesiophobia.

*P2: Before the dialysis, my health was already quite poor. Now that the surgery is over, I always feel weak and exhausted even when doing household chores. I always want to lie down.*

*P12: After I do some activity for a while, I feel my heart beating faster and I do not have enough energy either. I do not want to continue the activity.*

*P3: After the kidney transplant surgery, I lay in bed for over a week before getting up to move around. At that time, I had no muscles on my body and was extremely weak.*

### Increased sensitivity to postoperative discomfort

After kidney transplantation, patients are highly sensitive to their own discomfort due to the impact of the disease and medication. For instance, they are extremely sensitive to the pain at the surgical incision site in the kidney transplant area. In this study, kidney transplant patients may confuse their own muscle pain, arrhythmia and other post-transplantation discomfort symptoms with those related to the surgery. Most patients are worried that exercise will have an adverse effect on the transplanted kidney, and as a result, they exhibit a willingness and behavior to limit their own physical activities.

*P5: After the kidney transplant surgery, I experienced arrhythmia. The doctor said it was caused by heart disease. But I felt that after walking for a long time, my heart rate would increase. Now I’m even afraid to move around.*

*P8: After I exercised too much, I felt some pain at the incision site in my kidney area, my muscles were sore, and I also felt short of breath. I was worried that the exercise might damage the transplanted kidney.*

### Psychological factors

#### Excessive vigilance towards transplanted kidneys

After kidney transplantation, patients tend to pay excessive attention and cherish their transplanted kidneys. They are worried that physical activities might pull on the transplanted kidneys, afraid that the incision might split or cause infection, and unwilling to undergo dialysis again. Therefore, the patient exhibits high levels of sensitivity and tension, experiencing excessive alertness. This fear and worry interweave with each other, thereby exaggerating the risks that exercise might bring, and leading to avoidance of effective exercise rehabilitation behaviors.

*P1: Every time I do a little bit of movement, I always feel uncomfortable in the new kidney area, with a pulling sensation. I’m very afraid that the incision will split open, so I do not dare to exercise.*

*P13: Now I need to take immunosuppressant drugs. I also cannot go out to interact with others. What if I get infected? I’d better stay at home and rest instead!*

*P9: I always feel there is something foreign inside my body. Every time I move, I worry that the position of the transplanted kidney might shift. I have to check the abdominal incision every day.*

### Decrease in exercise self-efficacy

After kidney transplantation, patients are affected by multiple factors. Some of them may experience anxiety and confusion regarding the transplanted kidney and their future health conditions. As the psychological pressure of kidney transplant patients increases, along with related negative emotions such as uncertainty about the disease, this will seriously undermine the patients’ confidence in physical rehabilitation, thus leading to avoidance behaviors towards physical rehabilitation.

*P10: My current physical condition is that of a complete wreck. Just being able to stay alive is a blessing. What else can I do? I just prefer to lie down!*

*P11: I’m not in the mood for exercising. I’m under a lot of mental stress. I do not know when this kidney will reject, nor do I know how long I can live. I think I’m only suitable for resting.*

### Self-esteem and the sense of social stigma

Since kidney transplant patients need to take immunosuppressants for life after the surgery, to prevent early infections, doctors will instruct them not to have too much contact with the public within 6 months after the operation. However, this may affect the patients’ sense of social value and their social network relationships. Over a long period of time, it will lead to feelings of shame and inferiority in kidney transplant patients, which causes them to develop a mindset of fear and avoidance of physical activity.

*P4: The doctor instructed me not to have excessive contact with others within 6 months after the surgery. Now I cannot go to the gym with my friends anymore. My friends have not contacted me for a long time, and I feel very inferior.*

*P6: Previously, I was a cycling coach. The doctor said that I was prone to infection 6 months after the kidney transplant and told me not to engage in excessive social activities. I could not return to my previous job either. I truly felt that I was useless.*

### Supporting system factors

#### Insufficient guidance from medical staff

Some kidney transplant patients believe that medical staff pay more attention to the medication and dietary guidance for kidney transplant patients, but health education on exercise-related knowledge has not received sufficient attention. During this interview, some kidney transplant patients indicated that they were not clear about the different exercise methods, frequencies, precautions, and benefits at various stages after the surgery. After a kidney transplant surgery, due to the lack of awareness of the importance of exercise and the background of Chinese traditional culture, most people believe that after getting sick, they should mainly rest. Patients may neglect the significance of exercise and choose not to participate in active physical activities. This seriously affects their enthusiasm and initiative for exercise-based rehabilitation after the surgery.

*P7: The doctor told me that I could not return to work within 6 months after the surgery. I did not know how to exercise either, so I mainly stayed at home to rest.*

*P3: The doctor only told me to do some appropriate exercise, but he did not specify the exact type of exercise. I just did exercise by walking.*

*P8: I do not know what benefits exercise has for the recovery of the transplanted kidney. The nurse did not tell me either. My family members advise me to focus on rest, so I try to avoid doing much exercise.*

### Lack of companionship from family or friends

Patients undergoing kidney transplantation have experienced the related procedures of preoperative dialysis and surgery. They need significant encouragement and emotional support on a psychological level. Therefore, the presence of family and friends is of vital importance to the patients. In this study, some kidney transplant patients believed that they were most likely to contract infections within 6 months after the surgery. Therefore, they chose to reduce their outdoor activities and social interactions. Moreover, the absence of family members’ company led the patients to worry about the safety of exercising, thus resulting in an avoidance of physical activities.

*P10: The doctor said that the period immediately after the surgery is the most vulnerable to infection. Right now, I’m too scared to go out with my friends, so I do not do any sports either. Instead, I just do some simple housework at home.*

*P1: My family does not have time to accompany me, and I’m also afraid of getting into an accident by going for exercise alone, so I do not dare to do exercise.*

### Insufficient resources for sports rehabilitation

The postoperative rehabilitation of kidney transplant patients is a lengthy process. Besides paying attention to medication, diet, and related check-ups and follow-ups, the management of exercise rehabilitation after kidney transplantation also deserves attention. Some kidney transplant patients have pointed out that the hospitals in their respective regions have not established training centers for post-transplantation exercise rehabilitation. This has made it extremely difficult for kidney transplant patients to obtain professional health and exercise-related medical information and resources, seriously affecting their enthusiasm for exercise and decision-making behavior.

*P4: After I’m discharged from the hospital, I have to undergo regular follow-up visits. I do not know where I can get involved in the rehabilitation activities related to kidney transplantation, so I can only stay at home.*

*P12: It would be great if there were a center for post-transplantation exercise rehabilitation for kidney patients. Then I would have the confidence to do some exercise, but I’m afraid of it.*

### Environmental factors

#### Unfavorable weather conditions

Due to the influence of environmental factors, some kidney transplant patients are afraid that their immune system might be affected when the weather is bad, and therefore they are reluctant to go out for exercise. However, when they are indoors, they do not know what kind of exercise to do. They develop a fear of exercise due to the fear that the poor weather might affect the health of their transplanted kidneys.

*P11: It’s too cold in winter. I do not even want to go out to buy groceries, let alone do any exercise.*

*P13: The weather is very changeable, and I’m afraid I might catch a cold if I go out. But at home, I do not know what kind of exercise to do.*

## Discussion

It is of great significance to formulate a scientific and well-planned rehabilitation exercise program in the early stage after kidney transplantation. Based on the fear-avoidance theory, this study explored the influencing factors of kinesiophobia in renal transplant patients during the early postoperative period. The factors mainly included physiological, psychological, support systems and environmental factors. This provided a scientific basis for researchers to effectively formulate rehabilitation exercise intervention plans for kidney transplant patients.

### Attach great importance to the early identification and management of symptoms after transplantation

Patients undergoing kidney transplantation belong to a special group of people with chronic diseases. Their body functions are much lower than those of healthy individuals. After the surgery, to prevent rejection reactions, patients need to take immunosuppressants for life, which will lead to possible different symptom reactions in the patients. Studies have shown that there are various symptom reactions and experiences among Chinese kidney transplant patients after surgery. These symptoms are characterized by their complexity and non-linear changes, such as tremors in the hands and pain at the wound site (Hu et al., 2025). Some studies have also found that the incidence of fatigue among Chinese kidney transplant patients in the early postoperative period is 53.1% (Liu et al., 2024). Patients often fail to notice these symptoms in time, which may affect their postoperative recovery. Some researchers have pointed out that fatigue is directly negatively correlated with physical activity, and it indirectly affects the physical activity of kidney transplant patients through exercise fear and self-efficacy (Liu et al., 2021). In this study, some kidney transplant patients were concerned that exercise might have adverse effects on the transplanted kidney, which led to psychological and behavioral reactions of fear towards exercise. At the same time, various symptom responses would reduce the patients’ exercise ability, thus causing them to reject exercise. This is consistent with the qualitative research results of foreign scholars on the fear of exercise in patients after coronary artery stent implantation (Xu et al., 2025). Therefore, it is of utmost importance to effectively manage the fatigue and early postoperative discomfort symptoms of kidney transplant patients. Actively optimizing the patients’ physiological conditions may be the key to promoting changes in their physical activity behaviors. Currently, researchers have conducted interventions using the brief breathing relaxation method from a mindfulness perspective, which has effectively and simply alleviated various discomfort symptoms in kidney transplant patients (Lim et al., 2025). Some researchers have also utilized Enhanced Recovery After Surgery (ERAS) protocols to shorten the postoperative hospital stay for kidney transplant patients and facilitate their rapid recovery (Eltemamy et al., 2025). Therefore, healthcare researchers can adopt a multi-modal rehabilitation approach, focusing on and improving the post-operative discomfort experiences of kidney transplant patients, gradually enhancing their physical recovery, and ultimately achieving the goal of overcoming kinesiophobia.

### Establish a correct understanding of the disease and enhance one’s own exercise self-efficacy

This study found that psychological factors are one of the key factors contributing to kinesiophobia in renal transplant patients during the early postoperative period. The main manifestations include patients’ excessive concern about the function of the transplanted kidney, low self-efficacy, and feelings of stigma. This study found that if kidney transplant patients are overly concerned about their kidney function, it may lead to behaviors of fear of movement. Some researchers have also pointed out that after kidney transplantation, patients experience physical and mental discomfort in the early stage, and they overly focus on and become nervous about the transplanted kidney, fearing that their own behavior might have a negative impact on the kidney and require another dialysis session (Hu et al., 2024). Therefore, medical staff should help patients develop a correct understanding of the disease and reduce excessive concern about the transplanted kidney. This is of vital importance for the post-operative recovery of kidney transplantation. This study also revealed that kidney transplant patients with low levels of self-efficacy for exercise have a certain degree of prejudice towards exercise and lose confidence and motivation for exercise rehabilitation after the transplant surgery. This is consistent with the qualitative research results of other researchers on patients after coronary artery surgery (Chen et al., 2025). Previous quantitative studies have also indicated that the exercise self-efficacy of kidney transplant patients is affected to some extent by their fear of exercise and their physical activity (Zelle et al., 2016). The above research results suggest that healthcare workers should actively enhance the exercise self-efficacy of kidney transplant patients, encourage them to engage in moderate exercise, and reduce their fear of exercise. Furthermore, the kidney transplant patients interviewed in this study had restricted social networks due to the unstable immune system in the early stage after the transplant, which might cause psychological stress for the patients. A quantitative study found that the proportions of anxiety and depression among kidney transplant patients were 24.3 and 9.9% respectively, and it was pointed out that negative emotions would affect the patients’ creatinine levels and quality of life (Nassar et al., 2025). Previous qualitative studies have also indicated that after kidney transplantation, patients experience changes in their body image and self-esteem, and they may develop certain negative emotions (Akıncı and Varışoğlu, 2024). A study conducted among patients with ankylosing spondylitis revealed that high levels of kinesiophobia and negative emotions can affect the physical activity levels of these patients (Karaca et al., 2024). Currently, some researchers have pointed out that virtual reality technology can be beneficial in alleviating the fear of movement in patients with chronic back pain and accelerating their recovery process and prognosis (Wang et al., 2023). Therefore, it is of great significance to dynamically assess the psychological stress levels of kidney transplant patients in the early postoperative period, and through positive psychological intervention, effectively alleviate the negative emotions and improve the physical performance of these patients.

### Provide knowledge related to post-transplantation exercise, and offer family support systems and medical resources

This study has identified that the inadequacy of the support system is a factor contributing to kinesiophobia among kidney transplant patients. The three aspects of the inadequate support system are: insufficient guidance from medical staff, lack of companionship from family or friends, and scarcity of exercise rehabilitation resources. Previous researchers have pointed out that the knowledge level of kidney transplant patients in China is not optimistic, being at a medium level (Huang et al., 2024). This is consistent with the results of this study. In this study, kidney transplant patients have a high demand for knowledge related to transplantation, especially lacking knowledge about exercise. A qualitative study indicates that kidney transplant patients believe that medical staff have neglected their emotional and social needs. The patients are eager to receive health guidance on transplantation from the medical staff (Been-Dahmen et al., 2018). A study has shown that a multimodal follow-up care plan can help improve the physical endurance, cardiovascular health and muscle strength indicators of kidney transplant patients (Pape et al., 2025). In conclusion, medical staff should attach great importance to the health education on exercise knowledge for kidney transplant patients after surgery, including the types, frequency and methods of exercise, and actively guide them to engage in scientific exercise after the surgery. In terms of family support, this study found that the support from family members and friends has a positive effect on the exercise rehabilitation of kidney transplant patients, which is consistent with the results of previous studies. A study has shown that family support is beneficial in enhancing the medication compliance of kidney transplant patients after surgery and improving their adaptability in terms of physical and mental health (Hu et al., 2024; Doğan et al., 2025). Therefore, healthcare researchers can guide the family caregivers of kidney transplant patients to actively participate in the patients’ exercise plans, promote communication between the patients and their family members, in order to alleviate the patients’ fear of exercise and improve their physical condition. The results of this study indicate that there is a severe shortage of continuous exercise rehabilitation resources for patients after kidney transplantation in China after they are discharged from the hospital. Some researchers have pointed out that by guiding kidney transplant patients to participate in online activity clubs and receiving guidance from professionals as well as support from peers, it is beneficial to increase the patients’ interest in activities (Malo et al., 2024). Some researchers have also implemented customized exercise prescription plans, combined with remote medical monitoring, to facilitate the scheduled exercise of kidney transplant patients (Vecchiato et al., 2024). Therefore, in the future, China’s medical resources can be appropriately allocated with sufficient funds and personnel to establish exercise rehabilitation centers for kidney transplant patients. Through scientific exercise prescriptions provided by medical staff and exercise therapists, the lifespan of the transplanted kidneys can be extended.

### Provide indoor sports resources and enhance the enthusiasm for exercise

The results of this study suggest that post-transplantation exercise phobia in kidney transplant patients is influenced by climatic factors. As the researcher’s area is located in the northeastern part of China, and especially during winter when the weather is quite cold, the fear of exercise among kidney transplant patients in this region may have been influenced by the weather. However, other southern regions of China do not have a winter season, and the environment and temperature are relatively favorable. Therefore, this factor needs to be further verified when conducting research on kidney transplant patients in those other regions. Some kidney transplant patients believe that unfavorable climatic conditions will pose a threat to the health of the transplanted kidney, and therefore are reluctant to engage in outdoor exercise, which is consistent with the results of other researchers (Billany et al., 2022). Therefore, this study suggests that healthcare professionals can offer online intelligent exercise methods to kidney transplant patients, such as providing wearable exercise devices and designing instructional videos. Secondly, medical staff can also provide kidney transplant patients with indoor-friendly exercises such as Qigong, Tai Chi, and yoga, which are gentle forms of physical activities. This helps reduce the psychological pressure of outdoor exercise and achieves the goal of alleviating exercise anxiety.

This study has identified the influencing factors of kinesiophobia in kidney transplant patients, providing a new perspective for clinical medical staff to formulate precise management intervention plans. This suggests that in future research, intelligent intervention methods can be utilized to reduce the level of exercise fear among kidney transplant patients and enhance their confidence in exercising, in order to achieve the goal of continuous rehabilitation of the transplanted kidneys.

## Limitations

Currently, the innovation of this study lies in the application of qualitative research methods, exploring the influencing factors of kinesiophobia in the early postoperative period from the perspective of kidney transplant patients. This provides scientific support for researchers in the field of kidney transplantation to understand and formulate exercise-related nursing intervention measures. However, this study has certain limitations. Firstly, this study was conducted only in a tertiary grade A hospital in Northeast China. The limitation of sample size and quantity may restrict the interpretation of qualitative research results and also affect the general applicability of the research results. Secondly, the selection of research subjects was limited by age, and this result is not applicable to children and adolescents. Thirdly, the data of this study was collected through patients’ self-reports, which poses the risk of subjective bias. Fourthly, Since the kinesiophobia in patients undergoing kidney transplantation may change dynamically, future researchers should conduct longitudinal qualitative studies on the kinesiophobia in patients after kidney transplantation at different time points.

## Conclusion

The characteristics of kinesiophobia in patients after kidney transplantation present a complex and nonlinear changing process. The influencing factors include physiological functions, psychological conditions, support systems, and environmental factors. These factors interact with each other and jointly lead to the occurrence and progression of kinesiophobia in patients. Therefore, healthcare researchers should conduct early assessment of kinesiophobia in kidney transplant patients after surgery and identify the influencing factors. They should also combine intelligent management methods to formulate comprehensive intervention measures. The intervention measures can focus on providing training on sports-related knowledge, establishing sports resources and rehabilitation centers, offering mental health counseling and consultation, and developing intelligent sports support systems. By taking appropriate measures, medical staff can alleviate the fear and anxiety of kidney transplant patients regarding physical activity, achieve the rational allocation of medical resources, and ultimately promote the recovery of the transplanted kidneys.

## Data Availability

The original contributions presented in the study are included in the article/supplementary material, further inquiries can be directed to the corresponding author.
